# A patient with paraganglioma undergoing laparoscopic resection: A case report

**DOI:** 10.1002/ccr3.4145

**Published:** 2021-06-09

**Authors:** Hisamichi Yoshii, Hideki Izumi, Takuma Tajiri, Masaya Mukai, Eiji Nomura, Hiroyasu Makuuchi

**Affiliations:** ^1^ Department of Surgery Tokai University School of Medicine Isehara Japan; ^2^ Department of Pathology Tokai University School of Medicine Isehara Japan

**Keywords:** general surgery, neurosurgery, oncology

## Abstract

Paraganglioma is a very rare extraadrenal nonepithelial tumor. The number of cases of laparoscopic surgery in Paraganglioma is small and controversial. This study encountered a case of successful transperitoneal laparoscopic surgery for a 56‐mm paraganglioma in a 53‐year‐old female. Moreover, previous reports on laparoscopic surgery for paraganglioma are reviewed.

## Graphical abstract

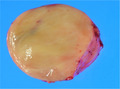

## 1 INTRODUCTION

Paraganglioma is a very rare extraadrenal nonepithelial tumor. The number of cases of laparoscopic surgery in paraganglioma is small and controversial. We experienced a successful case of transperitoneal laparoscopic surgery for 56 mm paraganglioma. In addition, previous reports of laparoscopic surgery for paraganglioma have been reviewed.

Paraganglia are groups of neuroendocrine tissues of neural crest origin closely related to the autonomous nervous system. A tumor derived from the paraganglia is a paraganglioma (PGL), which is an extraadrenal nonepithelial tumor.^1^ The standard treatment is surgical treatment. Moreover, the safety of laparoscopic surgery has been reported in recent years.

## 2 CASE EXAMINATION

A 53‐year‐old woman was diagnosed with an ultrasound image of her abdominal mass during a health examination. The patient was then referred to the hospital for a close examination of this abdominal mass. No past medical history and family history. Blood test findings: Hemoglobin, 14.0 g/dL; Carcinoembryonic antigen, 3.0 ng/mL; Carbohydrate antigen 19‐9, 6.7 U/mL; and Soluble interleukin‐2 receptor, 205 U/mL.

**FIGURE 1:**
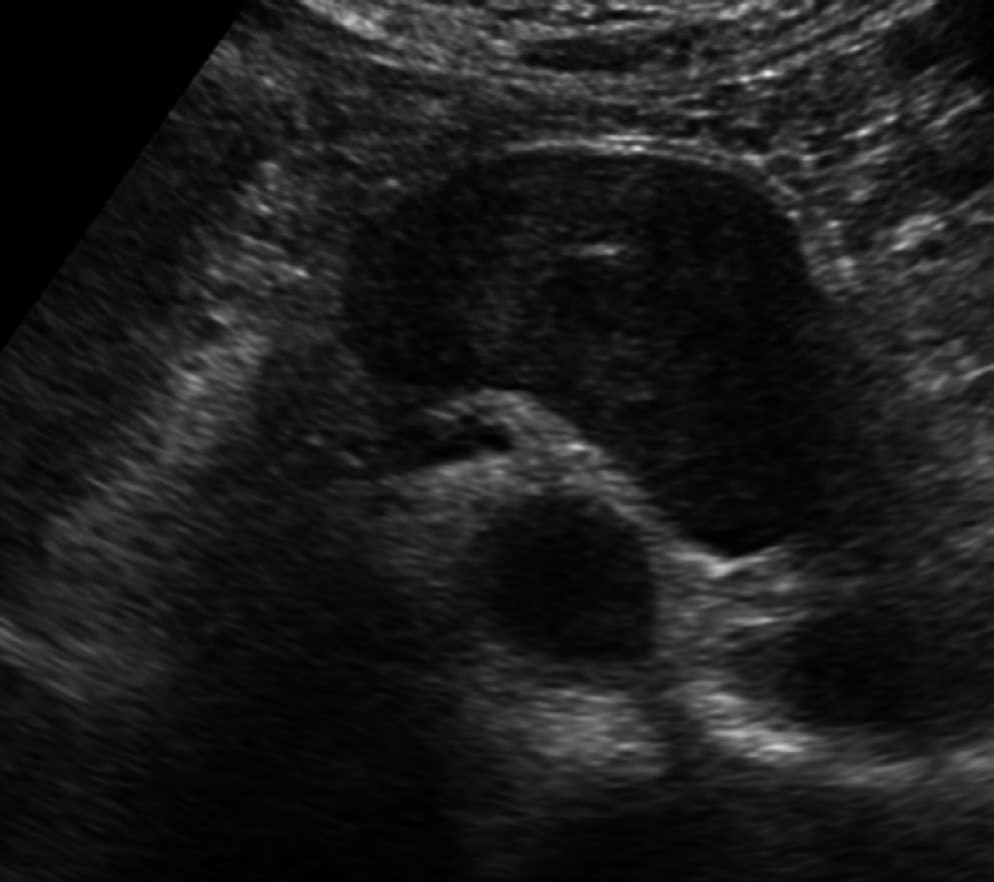
Abdominal ultrasonography image. A 56 × 43‐mm hypoechoic tumor with a smooth margin, internal heterogeneity, and no blood flow signal at the posterior wall of the gastric corpus and tail of the pancreas

**FIGURE 2:**
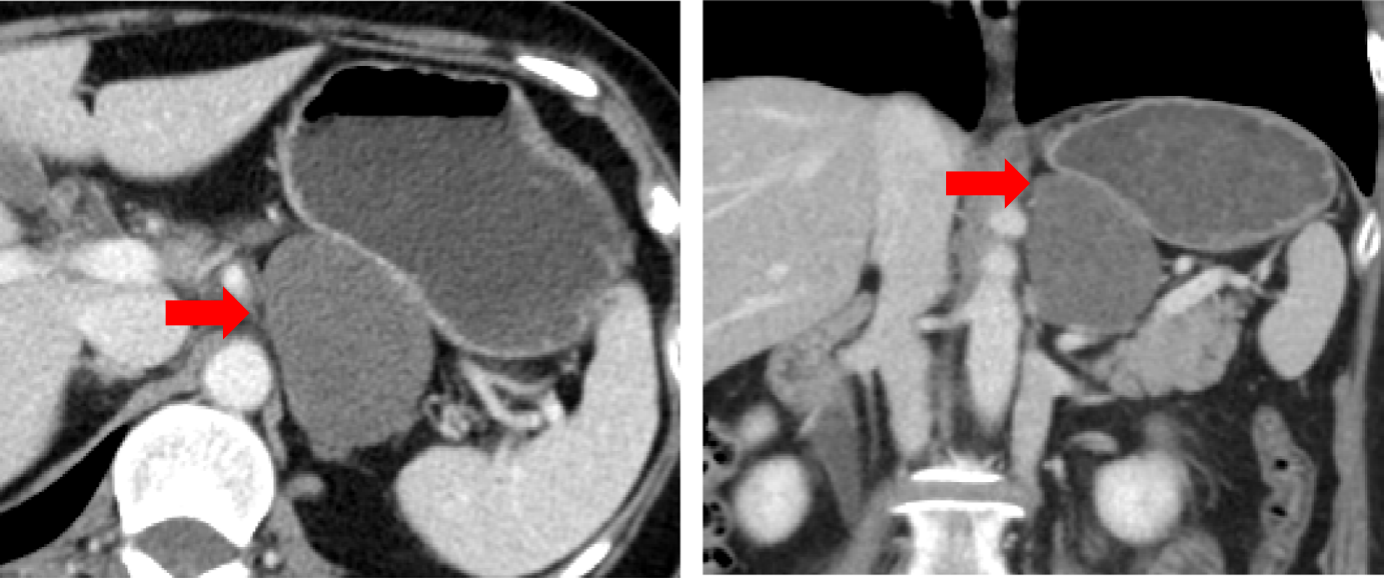
Abdominal contrast‐enhanced computed tomography images. A 56.7 × 37.9 × 54.7 mm between the posterior wall of the gastric corpus and the right margin of the abdominal aorta (red arrows)

**FIGURE 3:**
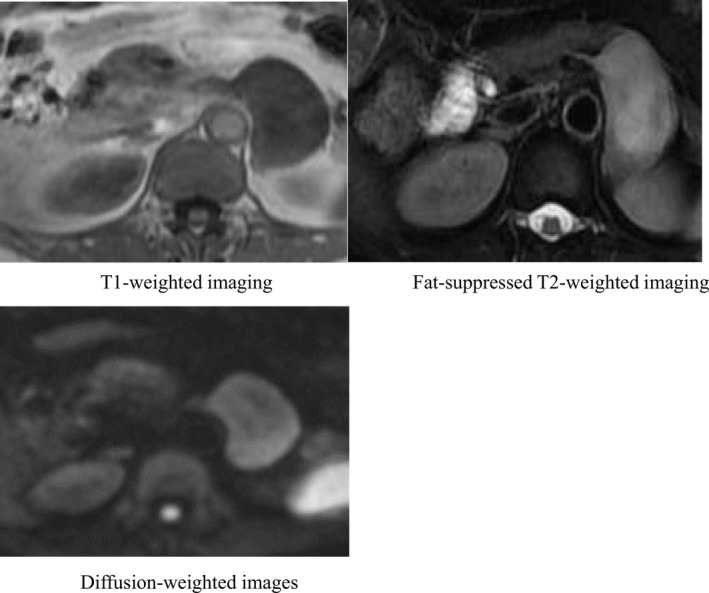
Abdominal contrast‐enhanced magnetic resonance images. Low signal on T1‐weighted imaging, faint and heterogenous high signal on T2‐weighted imaging, and faint high signal on diffusion‐weighted images. T1‐weighted imaging, Fat‐suppressed T2‐weighted imaging, Diffusion‐weighted images

**FIGURE 4:**
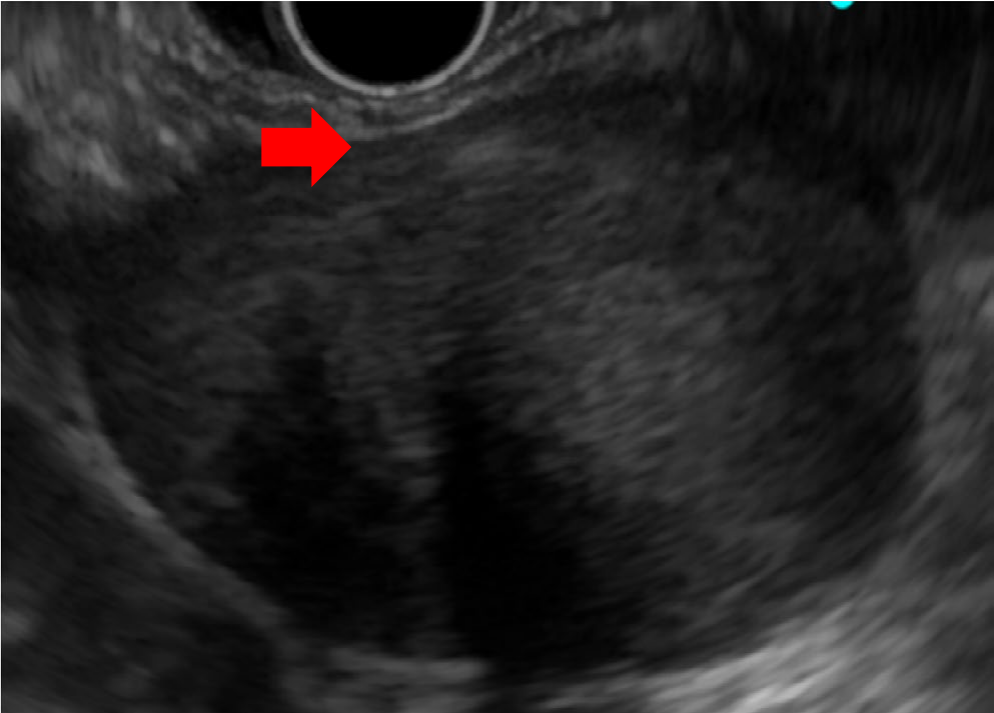
Endoscopic ultrasound image. A 47.3 × 31.3‐mm hypoechoic tumor at the posterior wall of the middle part of the gastric corpus. It is suspected to be derived from the muscular layer of the fourth layer of the gastric wall (red arrow)

Abdominal ultrasonography showed a 56 × 43 mm hypoechoic tumor with smooth edges, internal heterogeneity, and no blood flow signals observed in the gastric corpus and posterior wall of the tail of the pancreas (Figure 1). Abdominal computed tomography (CT) shows a low contrast of 56.7 × 37.9 × 54.7 mm between the posterior wall of the gastric corpus and the right edge of the abdominal aorta, with low contrast, smooth edges, and internal heterogeneity. A malignant tumor was observed. (Figure 2). On abdominal magnetic resonance imaging (MRI), T1, T2, and diffusion‐weighted images showed low signal, weak and nonuniform high signal, and weak high signal, respectively. Capsular fat components were not observed on fat‐suppressed T2‐weighted images. It may be continuous from the right edge of the aorta. However, the continuity with the gastrointestinal tract was not clear. Schwannoma and leiomyoma were considered as the diagnosis (Figure 3) Upper gastrointestinal endoscopy showed no clear extrinsic compression of the posterior wall of the body of the stomach. Endoscopic ultrasonography revealed a 47.3 × 31.3 mm hypoechoic tumor on the posterior wall of the middle body of the stomach. It is derived from the 4th muscular layer of the stomach wall (Figure 4). Diagnosis with fine‐needle aspiration was difficult due to insufficient amount of tissue.

### 2.1 Differential diagnoses

Based on the aforementioned findings, gastrointestinal stromal tumor (GIST) of the stomach, Schwannoma, and leiomyoma were listed as differential diagnoses. GIST of the stomach was most suspected, and the treatment modality was decided to be surgery.

**FIGURE 5:**
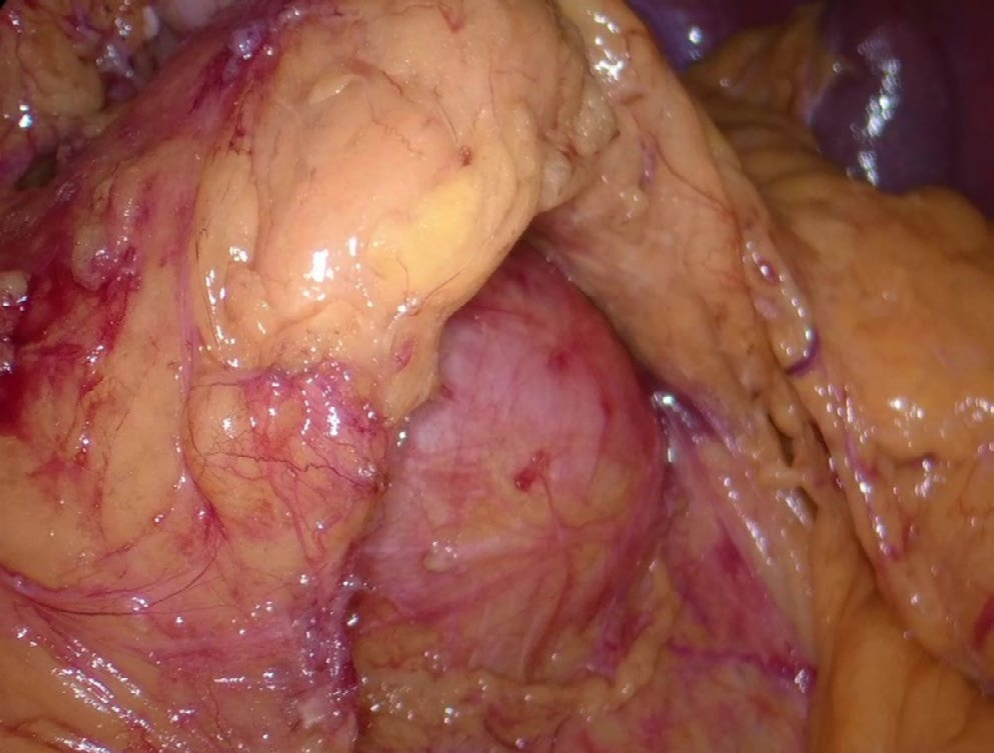
Surgical findings. The pancreas was tunneled and lifted; the tumor and the retroperitoneum were then exfoliated

**FIGURE 6:**
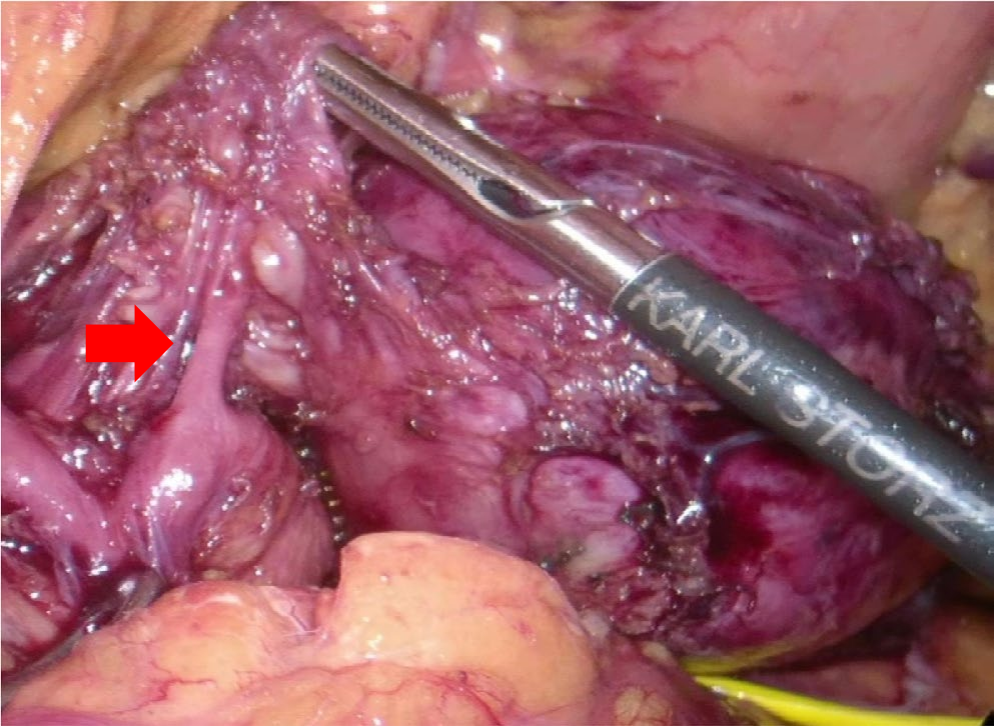
Surgical findings. Feeding vessels flow from the left gastric artery toward the tumor (red arrow)

Surgery was started in the lateral recumbent position with a transabdominal approach. Ports were inserted to form a reverse trapezoid with the umbilicus as the center. The tumor was separated from the gastric wall after the omental bursa was opened. The tumor was at a location surrounded by the left margin of the aorta, the upper margin of the renal artery, and the upper margin of the pancreas and the splenic hilum. The exfoliation of the retroperitoneum and the tumor was difficult. Thus, the pancreas was tunneled and lifted to achieve this (Figure 5). Feeding blood vessels were flowing from the left gastric artery to the tumor, so they were cut out and the tumor was removed (Figure 6). The procedure was a laparoscopic tumorectomy. The surgical duration was 247 min, and the hemorrhage volume was 10 mL. There were no changes in the intraoperative vital signs.

**FIGURE 7:**
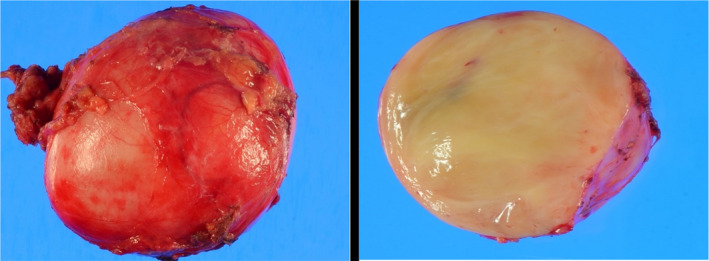
Macroscopic findings. A round tumor with capsule with a clear boundary was observed, and no necrosis existed on the cut surface. Moreover, yellow consolidation was observed

**FIGURE 8:**
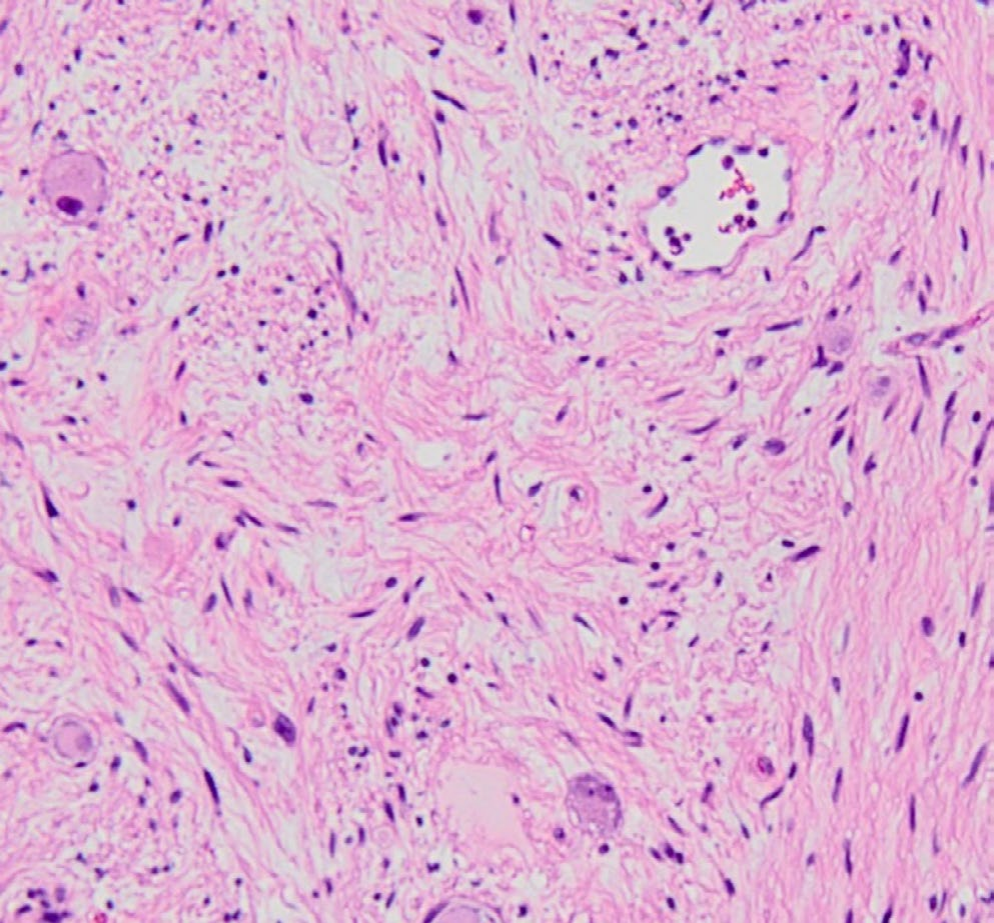
Histopathological findings. Supporting tissues and capillaries were observed around the pleomorphic tumor cells and alveolar aggregates, exhibiting a Zellballen pattern

**FIGURE 9:**
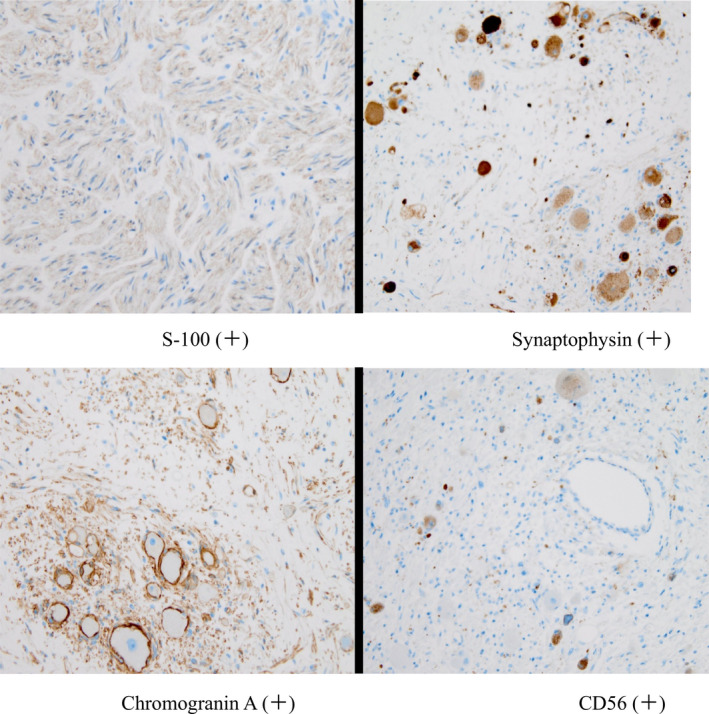
Immunohistochemistry staining. S‐100 (+), Synaptophysin (+), Chromogranin A (+), CD56 (+)

The macroscopic finding was a round tumor with a capsule with a clear boundary, and there was no necrosis on the cut surface. Moreover, yellow consolidation was observed (Figure 7). Histopathological findings showed supportive tissue and capillaries around polymorphic tumor cells and alveolar aggregate, showing a zellballen pattern. In addition, a large number of ganglion cells were observed (Figure 8). The following immunohistochemical findings were observed: S‐100 (+) in supporting cells, synaptophysin (Syn) (+) and CD56 (+) in ganglion cells, and chromogranin A (CgA) in ganglia. (+). The Ki67 index was <1% (Figure 9). The final diagnosis was PGL.

The postoperative course showed high levels of drain amylase and pancreatitis on the 3rd postoperative day. The patient improved with fasting and was discharged 12 days after surgery.

## 3 DISCUSSION

PGLs are currently clinically and biologically divided into two groups, based on the parasympathetic and the sympathetic nervous system involvement, according to the World Health Organization classification. PGLs arising from the parasympathetic ganglia mainly affect the head and neck. Thus, PGLs are described as two subgroups based on location, namely head and neck PGL and sympathetic PGL.^1^

Sympathetic PGLs, originating from the chest and abdominal sympathetic nerves, account for 80% of all PGLs.^2^ Moreover, 85% of sympathetic PGLs occur beneath the diaphragm and are particularly observed in the retroperitoneum around the adrenal and renal areas around the organ of Zuckerkandl and the bladder.^3^, ^4^ In addition, they are observed in the chest and heart.^5^, ^6^

PGL is usually characterized by catecholamine‐related symptoms, such as persistent/paroxysmal hypertension, diaphoresis, palpitation, headache, and anxiety neurosis.^7^ The sudden release of catecholamine makes it severe, with symptoms such as pulmonary edema, cerebral hemorrhage, hypertensive crisis, and cardiovascular disorders.^8^, ^9^, ^10^ However, about 10% of asymptomatic PGLs may be discovered with the advancements in imaging.^11^

Biochemical tests confirm the excessive secretion of catecholamine or metanephrine.^10^ Free metanephrine in the blood and urine is a specific marker for chromaffin tumors and is superior to catecholamine.^12^ In addition, blood metanephrine evaluation is superior to 24‐h urinary metanephrine evaluation in sensitivity and specificity.^13^ Furthermore, the test for the detection of urinary vanillylmandelic acid has the lowest sensitivity.^13^ PGL can be diagnosed with almost 100% certainty when metanephrine in the blood and urine is more than four times the normal upper limit.^13^ As this case was initially suspected as GIST of the stomach, blood and urine tests could not be performed before surgery.

The history rather than symptoms, such as high blood pressure and headaches, were considered to be nonfunctional.

In the imaging studies performed in the current case, contrast‐enhanced CT revealed consolidation with a contrast effect. Moreover, in MRI tests, T1‐ and T2‐weighted images showed low and high signals, respectively. ^123^I‐meta‐iodobenzylguanidine is a high‐sensitivity test and it is useful for adrenal tumors or metastases.^14^

Further, patients with PGL are also recommended to undergo genetic screening to detect genetic mutations that cause the disease. As many as 20 susceptibility genes have been currently discovered for PGL and pheochromocytoma. Germline mutations such as those in *RET*, *VHL, SDHA, SDHB, SDHC, SDHD, SDHAF2*, and *MAX* are related to PGL.^15^, ^16^ Patients with family history and below the age of 50 years are recommended to undergo genetic screening.^17^

Proper preoperative management becomes necessary if excessive secretion of catecholamine is confirmed before surgery. Preoperative preparation by administering α‐adrenergic blockers, β‐adrenergic blockers, or calcium channel blockers and communication between the anesthesiologist‐surgeon team during surgery is important.^17^

The standard treatment for PGL is surgical resection. A PubMed search for reports on laparoscopic surgery for abdominal PGL with paraganglioma/laparoscopic revealed 12 case reports, including this case, in a 10‐year period from 2010 to 2020 (Table 1).^18^, ^19^, ^20^, ^21^, ^22^, ^23^, ^24^, ^25^, ^26^, ^27^, ^28^ The average age was 44.9 years (22‐72), and the male:female ratio was 5:7. Laparoscopic approaches consisted of the transperitoneal approach in 11 cases and transthoracoabdominal approach in 1 case. Peritoneal approach was performed in the supine and lateral recumbent positions in six and five cases, respectively. No cases were found with the retroperitoneal approach of surgery. Tumor location was with a ratio of right:left = 7:4 at the aortic bifurcation in 1 case, above the renal artery in 6 cases, and under the renal artery in 6 cases. The average maximum tumor diameter was 52.5 mm (28‐82 mm), the average surgical duration was 177 min (100‐325 min), the average hemorrhage volume was 77.6 mL (little–340 mL), and the average duration of hospitalization was 5.2 days (2‐12 days). Only pancreatitis, in terms of complications, was noted in the present case. In addition, tumor location was compared above and below the renal artery, and the average surgical duration for cases with the tumor above the renal artery and below the renal artery was 208.8 and 130.6 min, respectively. The respective average hemorrhage volumes were 114.3 and 16.6 mL. The surgical duration was longer and the hemorrhage volume larger for tumors above the renal artery.

**TABLE 1:** Cases of laparoscopic surgery for abdominal paraganglioma performed during 2010‐2020 by PubMed search

No	Author	Age	Sex	Approach method	Tumor location	Maximum diameter of tumor (mm)	Surgical duration	Hemorrhage volume (mL)	Complication	Duration of hospitalization
1	The current case 2020	53	Female	Transperitoneal	Above the left renal artery	56	247	10	pancreatic fistula	12
2	Ahmed 2020^18^	23	Male	Transperitoneal Lateral recumbent position	Aortic bifurcation	50	NA	NA	None	NA
3	Xiamg 2020^19^	45	Male	Transperitoneal Lateral recumbent position	Under the right renal artery	72	120	50	None	5
4	Pietro 2020^20^	26	Male	Transperitoneal	Under the left renal vein	35	130	small amount	None	3
5	Antonios 2019^21^	69	Female	Transperitoneal	Under the right renal artery	45	142	small amount	None	3
6	Tomoaki 2019^22^	72	Male	Transperitoneal Lateral recumbent position	Above the right renal artery	70	231	200	None	NA
7	Hisataka 2018^23^	51	Female	Transperitoneal	Above the right renal artery	26	325	340	None	7
8	Mohammad 2018^24^	24	Female	Transperitoneal Lateral recumbent position	Under the left renal artery	35	NA	NA	None	5
9	Zar 2017^25^	26	Female	Transperitoneal	Above the right renal artery	82	120	40	None	5
10	Hrishikesh 2016^26^	22	Female	Transperitoneal Lateral recumbent position	Under the left renal artery	80	125	40	None	4
11	Yutaka 2015^27^	64	Male	Transperitoneal	Above the right renal artery	28	230	36	None	6
12	Altug 2010^28^	64	Female	Transthoracoabdominal Lateral recumbent position	Above the right renal artery	48	100	60	None	2

Pancreatitis was confirmed in the present case, and this is a point for reflection. Pancreatitis occurred because the pancreas had been tunneled for the dorsal tumor treatment. The retroperitoneal approach in the lateral recumbent position instead of the supine position was considered the best method. Ensuring accurate preoperative diagnosis, tumor location, and size and performing the surgery in the best position is necessary.

The safety and effectiveness of laparoscopic surgery for pheochromocytoma have been extensively reported. However, laparoscopic surgery for PGL is controversial. The comparison between laparoscopic surgery and laparotomy for PGL has been reported in a small‐scale experiment, and a decrease in hemorrhage volume and duration of hospitalization has been reported in this study.^29^ In addition, the comparative study between patients with pheochromocytoma and PGL undergoing laparoscopic surgery reported that laparoscopic PGL had a longer surgical duration. However, no significant difference existed in hemorrhage volume and duration of hospitalization.^30^ The selection of the laparoscopic approach is determined by the surgeon's preferences and skills, and the patient's physique, body mass index, tumor size, and location.^31^ Some reports have indicated that the retroperitoneal approach shortens the surgical duration.^32^, ^33^ In addition, reports also exist on the single‐site and robotic surgeries.^34^, ^35^

In terms of histological features, sympathetic PGLs and pheochromocytomas consisting of polygonal cells, called *chromaffin* cells, exhibit amphophilic to basophilic cytoplasm. Tumor cells are separated by the capillary plexus and arranged in an alveolar pattern (Zellballen architecture). Cytological characteristics include granular cytoplasm, prominent nucleoli, vesicular nuclei, pseudoinclusions inside nuclei, and so on. There may be secondary changes like hemorrhage, hemosiderin deposition, sclerosis, and pigmentation of the lipofuscin or melani.^2^, ^36^

Immunohistochemistry could confirm the pathological diagnosis and assist in making a differential diagnosis with other microscopically similar tumors. CgA is the most specific feature and helps distinguish PGLs from other neuroendocrine tumors. PGLs are usually positive for Syn, which is less specific than CgA because diffused positive Syn staining is also observed in adrenal cortical carcinomas.^37^, ^38^, ^39^ CD56 is also an important neuroendocrine marker.^40^ PGLs are usually negative for keratins. A Ki‐67 proliferation index >3% significantly predict the malignant potential and prognosis of PGLs.^41^

In addition, Pheochromocytoma of the Adrenal Gland Scaled Score (PASS), proposed by Thompson et al in 2002, is an index showing the malignancy potential of malignant pheochromocytomas.^42^ Malignant PGLs show a PASS score of ≥4. Of the 12 parameters of PASS, necrosis, capsular invasion, vascular invasion, cellular monotony, high mitosis, atypical mitotic figures, and nuclear hyperchromasia were significant predictors of malignancy.^43^ The tumor in this case had a Statistical Package for the Social Sciences score of 1 and was highly likely to be benign. However, malignant PGL has been reported to be 10%‐20% of the reported cases,^44^ and the malignancy risk is reported to be high despite young age and tumor size. Thus, strict follow‐up is important.^42^, ^45^

In conclusion, a laparoscopic tumorectomy by the transperitoneal approach was performed for a case of PGL. Laparoscopic surgery for PGL is generally not recommended. However, in the present case, it was considered to be completely safely resected after examining previous studies and reported cases. Fully understanding the preoperative diagnosis, tumor size, and location and performing the surgery with the best approach method and surgical position is important.

## CONFLICT OF INTEREST

None declared.

## AUTHOR CONTRIBUTIONS

HY: wrote this paper. TT: performed the autopsy, reviewed the pathological findings, and revised the manuscript. HI reviewed the medical image findings and helped to write the manuscript. MM, EN, and HM helped write the manuscript. All authors read and approved the final manuscript.

## ETHICS APPROVAL AND CONSENT TO PARTICIPATE

Declaration of Helsinki; Patient gave their informed consent prior to their inclusion in the study.

## CONSENT FOR PUBLICATION

Patient gave their informed consent prior to their inclusion in the study.

## Data Availability

Data sharing does not apply to this article because no datasets were generated or analyzed during the current study.
